# Analyses of locomotion, wing morphology, and microbiome in *Drosophila nigrosparsa* after recovery from antibiotics

**DOI:** 10.1002/mbo3.1291

**Published:** 2022-06-09

**Authors:** Simon O. Weiland, Matsapume Detcharoen, Birgit C. Schlick‐Steiner, Florian M. Steiner

**Affiliations:** ^1^ Department of Ecology University of Innsbruck Innsbruck Austria; ^2^ Division of Biological Science, Faculty of Science Prince of Songkla University Hat Yai Thailand

**Keywords:** *Acetobacter*, Proteobacteria, symbiosis, tetracycline, *Wolbachia*, 16S amplicon sequencing

## Abstract

Antibiotics, such as tetracycline, have been frequently used to cure arthropods of *Wolbachia* endosymbionts. After the symbionts have been removed, the hosts must recover for some generations from the side effects of the antibiotics. However, most studies do not assess the direct and indirect longer‐term effects of antibiotics used to remove *Wolbachia*, which may question the exact contribution of this endosymbiont to the effects observed. Here, we used the fly *Drosophila nigrosparsa* treated or not with tetracycline for three generations followed by two generations of recovery to investigate the effects of this antibiotic on the fly locomotion, wing morphology, and the gut microbiome. We found that antibiotic treatment did not affect fly locomotion two generations after being treated with the antibiotic. In addition, gut‐microbiome restoration was tested as a more efficient solution to reduce the potential side effects of tetracycline on the microbiome. There was no significant difference in alpha diversity between gut restoration and other treatments, but the abundance of some bacterial taxa differed significantly between the gut‐restoration treatment and the control. We conclude that in *D. nigrosparsa* the recovery period of two generations after being treated with the antibiotic is sufficient for locomotion, and suggest a general assessment of direct and indirect effects of antibiotics after a particular recovery time.

## Graphical abstract

We found no effect of tetracycline on locomotion in *Drosophila nigrosparsa* and differentiation of wing shape and differences in bacterial diversity between the control and gut‐restoration groups three generations after tetracycline treatment.

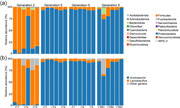

## 1 INTRODUCTION

Tetracycline is a broad‐spectrum antibiotic inhibiting bacterial protein synthesis by binding to the 30S ribosomal subunit. In arthropods, it is used to study the effect of endosymbionts *Wolbachia* (Ballard & Melvin, 2007). These Alphaproteobacteria infect 40%–60% of arthropod species and can have various effects on their hosts (Sazama et al., 2017; Zug & Hammerstein, 2012). However, tetracycline acts also on host enzymes and mitochondrial proteins by inhibiting the metabolism, synthesis, and repair of nucleic acids (Brodersen et al., 2000). In *Drosophila*, tetracycline has a negative effect on mitochondrial DNA density, mitochondrial metabolism (Ballard & Melvin, 2007), and host fitness (Miller et al., 2010). Therefore, after the antibiotic treatment, a recovery time prior to starting further experiments is important. Two generations have been reported as sufficient in *Drosophila* to reduce side effects of tetracycline on the host, such as changes in development (Fry et al., 2004; Harcombe & Hoffmann, 2004).

The composition of gut microbiota also changes with the antibiotic treatment (Jung et al., 2018; Raymann et al., 2017; Zhu et al., 2019; Zouache et al., 2009). Changes in the abundance of Proteobacteria (Pseudomonadota) and Firmicutes (Bacillota) in the microbiome after tetracycline treatment were described (Chao et al., 2020). Several studies have identified the importance of various bacterial taxa on their hosts, such as development (Buchon et al., 2009; Storelli et al., 2011), physiological processes, lifespan (Gilbert et al., 2018; Sommer & Bäckhed, 2013), disease resistance (Sansone et al., 2015), behavior (Selkrig et al., 2018), and gut morphology (Broderick et al., 2014).

This study aims to examine the effect of tetracycline on *Drosophila nigrosparsa* two generations after using tetracycline. To assess exclusively the effect of tetracycline on the insect and not that of the loss of *Wolbachia* resulting from the use of tetracycline, we use an uninfected population. For this purpose, noninfected *D. nigrosparsa* were treated with tetracycline and compared with a control to investigate the sole effect of the antibiotic on the flies. We investigated changes in larvae and adult locomotion, as well as adult wing morphology since both were included in a previous study examining the effects of *Wolbachia* on *D. nigrosparsa* (Detcharoen et al., 2020). The gut microbiome was characterized in this study during and after treatment with tetracycline, and in addition, gut microbiome restoration was tested as a solution to quickly reduce the potential side effects of tetracycline on the microbiome more quickly than without using it. Better knowledge of the long‐term effects of tetracycline on *D. nigrosparsa* is needed for better interpretation of published (Detcharoen et al., 2020) and future results. For example, there has been a recent focus on this alpine fly species for climate change research (Kinzner et al., 2019).

## 2 MATERIALS AND METHODS

### 2.1 Study system *D. nigrosparsa*

The distribution area of *D. nigrosparsa* is in Central and Western Europe. In Central Europe, the fly lives at about 2000 m above sea level (Bächli et al., 1985, 2004). The fly is well adapted to its extreme environment (Kinzner et al., 2016, 2018; Tratter Kinzner et al., 2019). Under artificial conditions at 19°C, the development time (embryo to adult) is around 60 days (Kinzner et al., 2016). No natural infection of *Wolbachia* in *D. nigrosparsa* is known (i.e., previous studies on the effect of *Wolbachia* infection in this species used transinfected flies; Detcharoen et al., 2020).

#### 2.1.1 Fly lines

**Figure 1:**
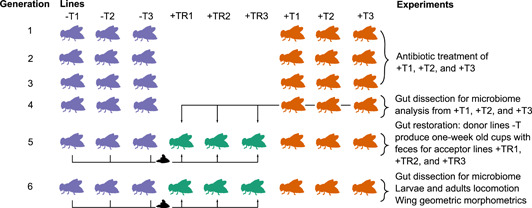
Chronological overview of the study using *Drosophila nigrosparsa*. Each fly line was kept in mating cages at a census size of 50 males and 50 females in every generation. Control lines −T1, −T2, and −T3 are fly lines not treated with tetracycline. Antibiotic‐treated lines +T1, +T2, and +T3 were treated with 0.05% tetracycline. Gut‐restoration lines +TR1, +TR2, and +TR3 were treated with feces from the control lines in generations 5 and 6.

*Drosophila nigrosparsa* was collected using fermented banana at Kaserstattalm in Stubai Valley, Tyrol, Austria (47.13°N, 11.30°E) in 2010 (Kinzner et al., 2018). There are no specific host plants for this species (Arthofer et al., 2016). The collected flies were used to establish the isofemale line iso12 by mating a single female and a single male to reduce genetic variation of the flies, and the offspring of this mating pair were inbred for 35 generations in small mating cages made of 300‐ml plastic cups (Cicconardi et al., 2017; Genomic Resources Development et al., 2015; Kinzner et al., 2018). The isofemale line used in this study was a subset of iso12 and was used in previous studies (Detcharoen et al., 2020, 2021). It was used to establish three control lines (not treated with antibiotics), namely, −T1, −T2, and −T3, and three antibiotic‐treated lines, namely, +T1, +T2, and +T3 (Figure 1). Gut‐restoration lines, +TR1, +TR2, and +TR3 were created by splitting the antibiotic‐treated lines in Generation 5 (i.e., two generations after tetracycline treatment). All flies were kept in mating cages (50 adult females and 50 adult males) (Kinzner et al., 2018) and supplied with grape juice agar, malt food, and yeast. Food was changed twice a week. Embryos or first‐stage larvae were transferred to glass vials with 8 ml malt food at a density of 80 embryos or 60 larvae per vial, respectively. All flies were kept at 19°C, 70% humidity, and a 16 h:8 h light:dark cycle.

#### 2.1.2 Antibiotic treatment

The antibiotic‐treated lines (+T1, +T2, and +T3) were treated with tetracycline hydrochloride (lot number SLBQ2368V, Sigma‐Aldrich) mixed in the malt food in a final concentration of 0.05% (D. I. Schneider et al., 2013) for three generations (Figure 1). Flies were fed with this mixed food at the larval and adult stage. After the treatment with tetracycline, these lines were fed regular malt food for another two generations.

#### 2.1.3 Gut microbiota restoration

In Generation 5, each of the antibiotic‐treated lines (+T1, +T2, and +T3) was divided to create gut‐restoration lines, namely +TR1, +TR2, and +TR3 (Figure 1). Individuals from the gut‐restoration lines were added to mating cages with feces from the corresponding control lines (i.e., line +TR1 was provided feces of line −T1). These cages were inhabited by flies from the control lines for one week. The cage was changed every week for two weeks.

#### 2.1.4 Larval locomotion

In Generation 6, 20 five‐day‐old larvae old were randomly collected from all lines for the locomotion experiment. Each larva was placed on a 55‐mm Petri dish filled with 2% (w/v) agarose and placed on a light pad (A4 Light Box, M. Way, China). The order of the larvae was chosen randomly. The locomotion of each larva was recorded for three min using a Sony XR155 Full HD video camera (Sony). The total crawling distance (mm) and mean speed (mm s^−1^) were analyzed with wrMTrck plugin version 1.04 (Nussbaum‐Krammer et al., 2015) in ImageJ version 1.53c (C. A. Schneider et al., 2012) with slight modifications (Brooks et al., 2016). The experiment was done over three days between 9:00 AM and 12:00 PM and was identical to that in Detcharoen et al. (2020). The locomotion data of the larvae were analyzed using a generalized linear mixed model (GLMM) with binomial and logit link function by using lines as a random effect nested within the treatment as a fixed factor with three levels (−T, +T, and +TR). The analysis was done using the package lme4 (Bates et al., 2015) in R version 4.0.3 (R Core Team, 2020) with an alpha = 0.05.

#### 2.1.5 Adult locomotion

Two methods were used for the adult locomotion experiments in Generation 6, the Rapid Iterative Negative Geotaxis (RING) assay (Gargano et al., 2005) and the *Drosophila* Activity Monitor (DAM5M) device (Trikinetics). RING allows for differentiation between walking and jumping, and DAM5 allows for assessing the number of moves over long periods. For each experiment, 20 two‐week‐old female flies of each line were randomly selected, anesthetized with CO_2_ for sexing three days before the experiment, and were put into separate vials with malt food without any further treatment. For both methods, flies were put at room temperature (19°C) for one h before the start of the experiments. The experiments took place between 9:00 AM and 1:00 PM.

For assessing walk and jump activities (RING experiment), the flies were transferred individually into heptane‐cleaned vials (100 × 24 × 1 mm, Scherf‐Präzision Europa) and clamped in random order in the RING apparatus. The fly‐containing vials were tapped quickly on the table, and the locomotion activities (walking and jumping) of flies were video recorded for three min with a video camera (Sony XR155 Full HD video camera, Sony). All fly lines were included in every run. The recorded videos were analyzed with ImageJ version 1.53c (C. A. Schneider et al., 2012). The instances of locomotion activities were counted manually. The experiment was identical to that in Detcharoen et al. (2020).

For the move‐activity experiment (DAM5M), each fly was transferred into a glass vial and placed in the DAM5M in random order. All fly lines were included in every run. The activities were measured every 20 s for an hour. The number of moves was detected automatically once a fly cross the infrared beam. The recorded data were then checked with DAMFileScan111X version 1.11 (Trikinetics). The statistical analyzes were the same as for the larvae.

#### 2.1.6 Wing morphology

**Figure A1:**
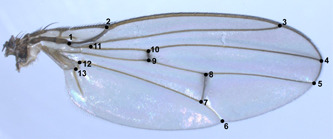
Thirteen landmarks on the wing of *Drosophila nigrosparsa* used to measure centroid size and shape.

To test the impact of tetracycline on wing morphology, 20 two‐week‐old female flies per line at Generation 6 were used. Wings were removed from each fly and stored in 96% ethanol. The stored left and right wings of each fly were put on a glass slide and covered with another slide. The upper and the lower side of the left and right wings of each fly were photographed using a Leica Z6 APO macroscope with a Leica MC190 HD camera with a 2.0x objective lens using the Leica Application Suite version 4.0 (Leica Microsystems). The wing photos were converted to a tps file using tpsUtil32 version 1.79 (http://www.sbmorphometrics.org/soft-utility.html). Thirteen landmarks were digitized manually using tpsDIG2w32 version 2.31 (http://www.sbmorphometrics.org/soft-dataacq.html) on every photo (Figure A1). The wing photos with the landmarks were analyzed with MorphoJ version 1.07a (Klingenberg, 2011). The landmarks were aligned by the principal axis. Images with incomplete landmarks were removed manually. The averages of shape and centroid size (i.e., square root of the sum of the squared distances of all landmarks from their centroid) from the upper and lower side of the wing from each fly were used for further analyses. Using the Procrustes ANOVA function implemented in MorphoJ, the potential imaging error between the lower and upper sides of the wing was accessed. Discriminant analysis between wings of all treatments was performed. Canonical variate analyses (CVAs) with 10,000 permutations were performed using regression residuals between centroid size and Procrustes coordinates. The regression residuals were used to remove variation among treatments that was caused by allometry. Principal components 1 and 2 of a principal component analysis were exported to R to calculate the analysis of similarity (ANOSIM) among treatments using the R package vegan version 2.5‐6 (Oksanen et al., 2019).

#### 2.1.7 Microbiome

Ten randomly chosen 14‐day‐old female flies were used per line. The antibiotic‐treated lines were examined for the first time in Generation 2, and all the lines of each treatment (control, antibiotic‐treated, and gut‐restoration) were checked in Generation 6. We did not analyze the microbiome of the control flies in Generation 2 because we assumed that all bacterial communities would be stable over time as they were reared in the same controlled environment. Each fly was killed in liquid nitrogen, surface‐sterilized using 2.5% bleach for 2.5 min, and washed twice with sterile MilliQ‐water, each for one min (Chandler et al., 2011), and the gut (crop, midgut, and hindgut) was removed under a stereomicroscope (SMZ800, Nikon) on a sterile slide with sterile forceps. Five guts from the same line per replicate were transferred into a sterile 1.5 ml microcentrifuge tube, two replicates per line. The guts were homogenized manually with sterile plastic pestles. Mock microbial community cells and DNA standards (Zymo Research) were used to check for DNA extraction efficiency and sequencing errors, respectively. One blank sample was used to check for bacterial contamination in the DNA extraction kit. The DNA was extracted with QIAamp DNA Mini Kit (QIAGEN), and the DNA was resuspended in sterile water. Human DNA contamination was checked using Alu J primer (Cannas et al., 2009) with quantitative PCR. The extracted DNA was amplified with bacterial 16S V3‐V4 region of ribosomal DNA universal primer 341F and 805R (Herlemann et al., 2011). The samples were sequenced with Illumina MiSeq. 2 × 300 bp using a single lane at IGA Technology Services (Udine). The Qiime2 pipeline version 2020.6 (Bolyen et al., 2019) was used for sequence analyses. DADA2 (Callahan et al., 2016) implemented in Qiime2 was used to trim the sequences, merge forward and reverse reads, and remove chimeras. The sequences found in the blank control were removed from all other samples.

SILVA release 138 (Quast et al., 2013) was used to assign taxonomy at the level of amplicon sequence variance (ASV). Alpha (i.e., bacterial richness of each sample) and beta diversity (i.e., bacterial communities among samples) were analyzed. To estimate alpha diversity using Faith's phylogenetic diversity, samples were rarefied to the minimum abundance found among them. The Kruskal–Wallis test with Benjamini & Hochberg correction for multiple comparisons was used to test for significant differences in alpha diversity among treatments. For beta diversity, nonmetric multidimensional scaling (NMDS) and ANOSIM based on ASVs at the species level were used. Both ANOSIM and NMDS use distance matrices for analysis; ANOSIM compares the variation within treatments with that among treatments and results in a global *R*‐value ranging from 0 to 1. Combining NMDS and ANOSIM is useful in that the visual and the numerical results can be interpreted together. Differential abundance at the bacterial species level between the three treatments was performed based on normalized read counts with geometric mean in the R package DESeq2 version 1.30.0 (Love et al., 2014). Benjamini‐Hochberg correction for multiple comparisons was used to adjust the *p*‐value for each ASV.

## 3 RESULTS

### 3.1 Locomotion

**Figure 2:**
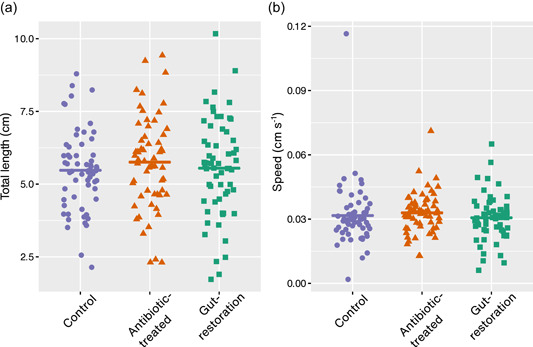
Each dot represents the total length (a) and speed (b) of movements of larvae of *Drosophila nigrosparsa* for three min (*N* = 60 for each treatment). Control, antibiotic‐treated, and gut‐restoration treatments are shown in purple, orange, and green, respectively. Plots show different y‐scales. Horizontal lines indicate means.

In larval locomotion, all treatments had similar crawling speed and distance (Figure 2, Table A1), and there were no significant differences among treatments regarding mean speed and distance (GLMM, speed, *χ* ^2^ = 2.23, *p* = .90; distance, *χ* ^2^ = 2.23, *p* = .90).

**Figure 3:**
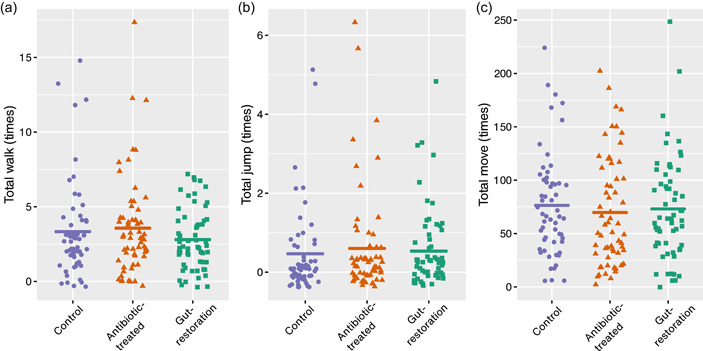
Walk (a) and jump (b) activities during three min of adult female *Drosophila nigrosparsa* from the rapid iterative negative geotaxis experiment. The number of moves (c) every 20 s for an hour of adult female *D. nigrosparsa* using the DAM5M method. Control, antibiotic‐treated, and gut‐restoration treatments are shown in purple, orange, and green, respectively (*N* = 60 for each treatment). Plots show different *y*‐scales. Horizontal lines indicate means.

In the walk and jump activities (RING locomotion assays), flies of all treatments on average walked around three times and jumped 0.5 times in three min (Figure 3, Table A1), which resulted in no significant difference among treatments (GLMM, walk, *χ* ^2^ = 10.30, *p* = .11; jump, *χ* ^2^ = 4.24, *p* = .64).

In the move activity (DAM5M locomotion assays), the control moved the most, followed by gut‐restoration and antibiotic‐treated treatments. However, no significant difference among treatments was found (GLMM, *χ* ^2^ = 2.23, *p* = .90).

### 3.2 Wing morphology

We removed 42 outliers of the initial 822 wing photos because of incomplete landmarks on the wings. The mean squares of imaging error were very low for both centroid size and shape (1.05 and 2.91 times lower than individual by side interactions for centroid size and shape, respectively).

No significant differences in size and shape were found between left and right wings of the flies of the control treatment (size *F*_1,4_ = 0.07, *p* = .80; shape *F*_22,88_ = 0.13, *p* = 1.00), the antibiotic‐treated treatment (size *F*_1,4_ = 0.00, *p* = .96; shape *F*_22,88_ = 0.08, *p* = 1.00), and the gut‐restoration treatment (size *F*_1,4_ = 0.04, *p* = .85; shape *F*_22,88_ = 0.22, *p* = 1.00).

**Figure A2:**
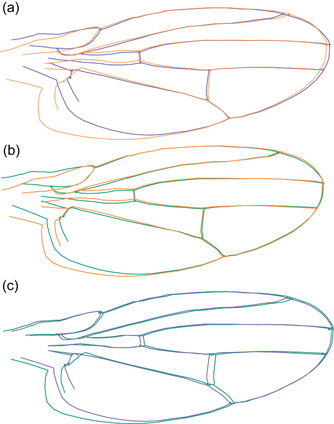
Wing shape differences between (a) antibiotic‐treated (orange) and control (purple), (b) antibiotic‐treated and gut‐restoration (green), and (c) control and gut‐restoration treatments calculated using discriminant analysis. All differences were magnified five times.

Mean wing shape was significantly different among the three treatments (Procrustes ANOVA; shape, *F*_44,132_ = 1.80, *p* = .006) and in all pairwise comparisons of treatments (antibiotic‐treated and control, antibiotic‐treated and gut‐restoration, and gut‐restoration and control; discriminant analysis, *p* < .001 in all comparisons; Figure A2), but centroid size was not. The Mahalanobis distance (distance between two treatments in multivariate space) was 5.07 between control and antibiotic‐treated treatments, 2.93 between control and gut‐restoration treatments, and 2.74 between antibiotic‐treated and gut‐restoration treatments.

**Figure 4:**
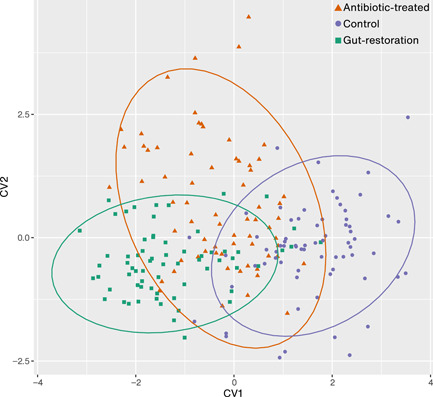
Canonical variate analysis with 10,000 permutations of wings of control (purple), antibiotic‐treated (orange), and gut‐restoration (green) treatments. Each dot represents an individual *Drosophila nigrosparsa* female at Generation 6 (*N* = 780). 95% confidence ellipses are shown.

The significant difference in wing shape among treatments we found, however, was not clearly projected by CVA. CVA showed that the gut‐restoration treatment overlapped more with the antibiotic‐treated treatment on the first axis, and the antibiotic‐treated treatment overlapped more with the control on the second axis (Figure 4). The ANOSIM statistic *R* values were very low and not significant in any instance: between antibiotic‐treated and control treatments, *R* was less than 0.01 (*p* = .12); between antibiotic‐treated and gut‐restoration treatments, *R* was 0.01 (*p* = .08); and between control and gut‐restoration treatments, *R* was less than 0.01 (*p* = .57).

### 3.3 Microbiome

**Figure A3:**
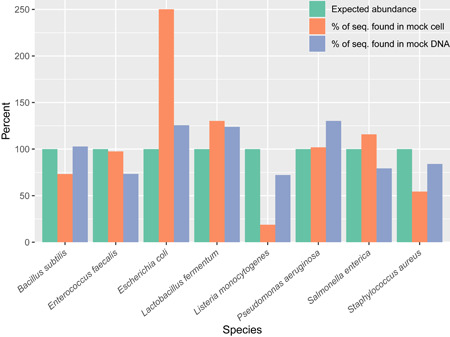
Positive control with the percentage of sequences of a mock cell (orange) and DNA (gray) communities (ZymoBIOMICS™, USA) from 16S sequencing compared with the expected results.

After trimming, the forward and the reverse sequences were 280 and 220 bases long, respectively. A minimum merged read was 37,034 from the blank sample and a maximum of 390,048 reads from the −T3 sample of Generation 6. The cell and DNA mock communities revealed a minor extraction and sequencing error. The mock cell extraction deviated by a total of 5% and 1% from the relative abundance of the mock cell and mock DNA community standards, respectively (Figure A3).

**Figure 5:**
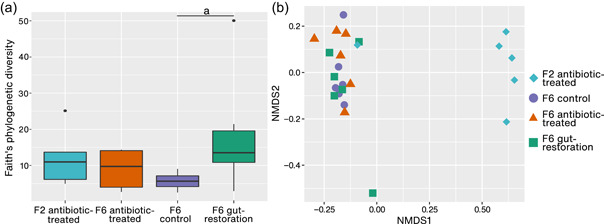
(a) Faith's phylogenetic diversity of all gut samples of *Drosophila nigrosparsa* in Generation 2 of antibiotic‐treated treatment and Generation 6 of antibiotic‐treated, control, and gut‐restoration treatments. (b) Nonmetric multidimensional scaling of samples at the species level. Antibiotic‐treated treatment in Generation 2 (turquoise) and Generation 6 (orange), control treatment in Generation 6 (purple) and gut‐restoration treatment in Generation 6 (green). Plots have different *x*/*y*‐scales. The letter indicates a significant difference.

There was some variation in alpha diversity among treatments; for example, the control had lower diversity than the others. Alpha diversity was significantly different between control and gut‐restoration treatments in Generation 6 (Kruskal–Wallis, *p* = .04) but not for other comparisons (control and antibiotic‐treated: *p* = .42; and between antibiotic‐treated and gut‐restoration: *p* = .42) (Figure 5a).

Beta diversity using NMDS based on ASVs showed a separation between the antibiotic‐treated samples from Generation 2 and all the samples from Generation 6 (ANOSIM *R* = 0.74) (Figure 5b). Yet, samples of all treatments in Generation 6 were more similar to each other (ANOSIM *R* = −0.003).

**Figure 6:**
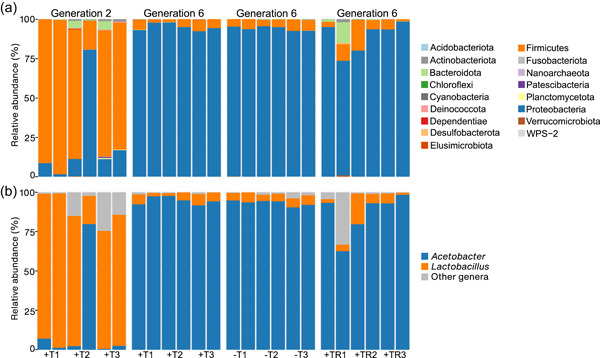
Relative abundance of bacterial phyla. Each replicate was a pool of five guts; two replicates per line were used. Antibiotic‐treated treatment Generations 2 and 6 (+T1, +T2, and +T3), control treatment Generation 6 (−T1, −T2, and −T3), and gut‐restoration treatment Generation 6 (+TR1, +TR2, and +TR3) are shown. Plots have different *y*‐scales.

**Figure A4:**
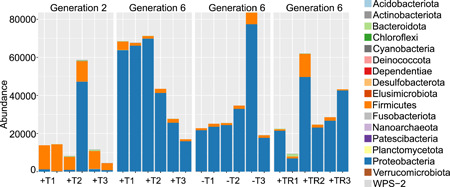
Absolute abundance of bacterial phyla. Each replicate was a pool of five guts; two replicates per line were used. Antibiotic‐treated treatment in Generations 2 and 6 (+T1, +T2, and +T3), control treatment in Generation 6 (−T1, −T2, and −T3), and gut‐restoration treatment in Generation 6 (+TR1, +TR2, and +TR3) are shown. Plots have different *y*‐scales.

*Lactobacillus* (phylum Firmicutes) were the most dominant bacteria of the antibiotic‐treated treatment in Generation 2. After recovering, *Acetobacter* became the most dominant genus in the antibiotic‐treated treatment, whereas *Lactobacillus* was the second most abundant (Figures 6 and A4). There was no genus that had the same abundance across treatments.

In Generation 6, several *Acetobacter malorum* ASVs had a significantly higher abundance in the antibiotic‐treated and gut‐restoration treatments than in the control treatment (Table 1). In contrast, ASVs of Enterobacteriaceae sp., *Enterococcus* sp., *Escherichia*‐*Shigella* sp., and *Staphylococcus* sp. had significantly lower abundances in the antibiotic‐treated and the gut‐restoration treatments compared with the control treatment. No significant difference in taxon abundance between the antibiotic‐treated and the gut‐restoration treatment was found. When comparing the antibiotic‐treated treatments of Generations 2 and 6, several bacterial taxa (but most strongly *Lactobacillus*) had significantly higher abundances in Generation 2 (while being treated with the antibiotic) than in Generation 6 (after recovery).

**Table 1:** Differential abundance of bacterial species between the antibiotic‐treated treatment (+T) and the control (−T) in Generation 6, between the gut‐restoration treatment (+TR) and the control (−T) in Generation 6, and between the antibiotic‐treated treatment (+T) of Generations 2 and 6 analyzed using the R package DESeq2.

Treatment or generation	ASV number	Species	Mean normalized read counts	*p*‐value	Treatment or generation with a higher abundance
+T and −T	14	*Acetobacter malorum*	791.70	<0.001	+T
	18	*A. malorum*	658.19	<0.001	+T
	19	*A. malorum*	596.39	<0.001	+T
	22	*Escherichia*‐*Shigella* sp.	14.85	<0.001	−T
	30	*Staphylococcus* sp.	8.36	<0.001	−T
	23	*Escherichia*‐*Shigella* sp.	8.08	<0.001	−T
	24	*Enterobacteriaceae* sp.	7.21	<0.001	−T
	25	*Enterococcus* sp.	5.89	<0.001	−T
	26	*Staphylococcus* sp.	4.20	<0.001	−T
+TR and −T	14*	*A. malorum*	282.75 (258.96)	<0.001	+TR
	18*	*A. malorum*	231.93 (231.93)	<0.001	+TR
	19*	*A. malorum*	204.24 (187.09)	<0.001	+TR
	35	*Lactobacillus fermentum*	22.72	<0.001	−T
	144	*Rhodobacteraceae* sp.	21.12	<0.001	−T
	32	*L. fermentum*	18.85	<0.001	−T
	30*	*Staphylococcus* sp.	12.25 (10.19)	<0.001	−T
	23*	*Escherichia*‐*Shigella* sp.	11.84 (9.84)	<0.001	−T
	24*	*Enterobacteriaceae* sp.	10.56 (8.78)	<0.001	−T
	25*	*Enterococcus* sp.	8.63 (7.18)	<0.001	−T
	26*	*Staphylococcus* sp.	6.15 (5.11)	<0.001	−T
	28	*L. fermentum*	5.31	<0.001	−T
+T between Generations 2 and 6	11	*Lactobacillus plantarum*	5983.09	0.006	Generation 2
	13	*L. plantarum*	4832.13	0.006	Generation 2
	15	*Lactobacillus brevis*	3993.30	0.025	Generation 2
	17	*L. plantarum*	3686.32	0.006	Generation 2
	16	*L. plantarum*	2953.96	0.008	Generation 2
	20	*L. brevis*	2013.58	0.025	Generation 2
	31	*L. brevis*	940.45	<0.001	Generation 2
	14	*A. malorum*	926.59	<0.001	Generation 6
	18	*A. malorum*	761.47	<0.001	Generation 6
	19	*A. malorum*	693.60	<0.001	Generation 6
	68	*Prevotella paludivivens*	93.95	0.005	Generation 2
	515	*Sulfitobacter* sp.	24.02	0.022	Generation 2
	514	*Pelomonas* sp.	24.02	0.022	Generation 2
	145	*Sulfitobacter* sp.	19.58	0.025	Generation 2
	165	*Rhodobacteraceae* sp.	17.87	0.025	Generation 2
	195	*Octadecabacter* sp.	15.89	0.027	Generation 2
	79	*Sulfitobacter* sp.	5.74	<0.001	Generation 2

*Note*: The treatment or generation that had the higher mean abundance among the treatments or generations compared is shown for each taxon. No bacterial taxa abundance was significantly different between the gut‐restoration treatment and the antibiotic‐treated treatment. Once the sample with the highest alpha diversity of the gut‐restoration treatment was removed, there was no change in the results between the gut‐restoration and the antibiotic‐treated treatments but the gut‐restoration treatment and the control (significantly different taxa are marked with asterisks, and the mean normalized read counts are given in parentheses).

We note that one sample of the gut‐restoration treatment had an outstandingly high alpha‐diversity value (Figure 5a). When removing this sample, the significant difference in alpha diversity between the gut‐restoration treatment and the control disappeared (Kruskal–Wallis, *p* = .07), and taxa with significantly different abundances between the gut‐restoration treatment and the control decreased from twelve to only eight; however, still, no significant difference between antibiotic‐treated and gut‐restoration treatments arose (Table 1).

## 4 DISCUSSION

We found significant differences in wing shape among treatments (control, antibiotic‐treated, and gut‐restoration) but no significant effect of tetracycline on larval and adult locomotion of *D. nigrosparsa* after two generations of recovery. When removing one sample of the gut‐restoration treatment that had an outstandingly high value of Faith's phylogenetic diversity (see further down on whether or not this may be justified), the restoration method could be considered successful as there were no significant differences in alpha diversity among treatments. In any case, some taxa still differ significantly between the gut‐restoration treatment and the control also after potential removal of that sample.

No locomotion activity of larvae and adults differed among the three treatments. This result indicates that the locomotion activities of flies two generations after recovery were not affected by tetracycline treatment. As we did not find any significant difference between the control and the antibiotic‐treated treatments, we can confirm that our previous results on locomotion that *Wolbachia*‐infected flies had higher locomotion activities than antibiotic‐treated flies (Detcharoen et al., 2020) were due to a direct effect of *Wolbachia*. We are not aware of the impacts of antibiotics on arthropods, but a recent study found that removing the gut microbiome via antibiotics induced changes in behavior in male mice. The changes, however, disappeared once the microbiome was restored (Vicentini et al., 2021).

We found that wing samples of each treatment were grouped with some overlap (Figure 4), and the mean shape of each treatment was significantly different when compared with another treatment. One of the potential reasons for the grouping of our samples may include a founder effect (i.e., the loss of genetic variation when a small subset of a large population establishes a new population). This effect can be observed after a few generations following the separation of flies, like in previous studies in *D. nigrosparsa* (Detcharoen et al., 2020) and *D. subobscura* (Santos et al., 2012, 2013). However, a founder effect appears unlikely here, as not only the treatments but also the lines have been separated for six generations (Figure 1). Although genetic variation is highly reduced in *Drosophila* isofemale lines, morphological differences can persist (Bubliy et al., 2001; Carreira et al., 2006). Another hypothesis for the morphological changes in the wings could be differences in the microbiome because, as demonstrated in *D. melanogaster*, gut bacteria influenced gut morphology through changes in the renewal rate and composition of cell types of the epithelium (Broderick et al., 2014). Yet, to our knowledge, there is no evidence that specific bacteria, such as *Acetobacter*, can influence wing morphology.

In the microbiome analysis of flies of Generation 6, we found some variation in alpha diversity among treatments. The significant differences between the control and the gut‐restoration treatment we observed were mainly from variation in our samples, which might indicate that the restoration process was not successful. Even though we report on the comparative analyses of alpha diversity with and without the one sample with outstandingly high Faith's phylogenetic diversity, we prefer to include all samples in the analyses as removing any sample would require a reason for doing so such as any known artifact. We are not aware of any such issue, and all samples were treated the same way. One possible reason for the variation in alpha diversity could be that the feces we used via the restoration method may have changed the *Acetobacter* abundance of the gut‐restoration treatment such as via competition of bacteria during the recolonization process. The high diversity in the gut‐restoration treatment we found was also observed in recolonized soil samples (Kaminsky et al., 2021); in that study, beta diversity also indicated that the microbiomes of these samples were similar to each other.

The abundance of several bacterial taxa such as *Lactobacillus* (Firmicutes) and *Acetobacter* (Proteobacteria) of the antibiotic‐treated treatment was significantly different between Generations 2 and 6. *Lactobacillus* and *Acetobacter* had the highest relative abundance during and after the antibiotic treatment, respectively. There was no genus with the same abundance across treatments as another one. Thus, taxa not influenced by antibiotics or antibiotics plus restoration were potentially resistant to the antibiotic, but not as competitive as *Acetobacter*. A decrease in Proteobacteria and an increase in Firmicutes during antibiotic treatment were observed in earthworms (Chao et al., 2020) and rats (Yin et al., 2015) treated with tetracycline. One explanation for an increase in the relative abundance of Firmicutes, including several *Lactobacillus* species, during tetracycline treatment, is that Firmicutes bacteria have higher resistance to tetracycline than other bacteria because they have a high number of tetracycline‐resistance genes (Berglund et al., 2020; Campedelli et al., 2019; Kobashi et al., 2007). However, tetracycline treatment does not always lead to an increase in Firmicutes, such as in the small brown planthopper *Laodelphax striatellus* (Zhang et al., 2020). In addition, the decrease of *Lactobacillus* and the increase of *Acetobacter* in generation 6 might be due to competition between them (Wong et al., 2015).

The significant differences in the abundances of some bacterial taxa between the control and the antibiotic‐treated treatment in Generation 6 mean that two generations after the last antibiotic treatment might not be enough for the gut microbiome to recover, and the differences between the control and the gut‐restoration treatment suggest that the gut microbiota of the gut‐restoration samples possibly was not fully restored (Table 1). Nevertheless, the absence of a significant difference in alpha diversity suggests that, apart from the differences in differential abundance, the restoration method was successful. The restoration method we used here has been used in some studies, but the microbiome of the flies after restoration was not checked (Baião et al., 2019; D. I. Schneider et al., 2019).

In summary, we found a significant difference in wing shape among treatments, and there were no significant differences in neither larval nor adult locomotion. There were some differences in alpha diversity and abundances of the gut microbiome among treatments. We cannot make a clear statement that the difference in wing morphology we found was an effect of differences in the microbiome as we lack firm evidence, but we can conclude that there was no effect of tetracycline on locomotion activities of *D. nigrosparsa* after two generations of recovery. Assessing the effect of a tetracycline treatment lasting more than three generations would be a good test of the potential effects of antibiotics on wing morphology. In any case, our study illustrates the importance of assessing both direct and indirect effects of any antibiotic after a particular recovery time, before or while assessing the effect of *Wolbachia* infection.

## AUTHOR CONTRIBUTIONS

**Simon O. Weiland**: Data curation‐Equal, Formal analysis‐Equal, Writing—original draft‐Lead, Writing—review & editing‐Equal. **Matsapume Detcharoen**: Data curation‐Equal, Formal analysis‐Equal, Writing—original draft‐Equal, Writing—review & editing‐Equal. **Birgit C. Schlick‐Steiner**: Conceptualization‐Equal, Formal analysis‐Equal, Funding acquisition‐Equal, Project administration‐Equal, Supervision‐Equal, Writing—review & editing‐Equal. **Florian M. Steiner**: Conceptualization‐Equal, Formal analysis‐Equal, Funding acquisition‐Equal, Project administration‐Equal, Supervision‐Equal, Writing—review & editing‐Equal.

## CONFLICT OF INTEREST

None declared.

## ETHICS STATEMENT

None required.

## Data Availability

Sequences are available in GenBank, BioProject accession number PRJNA694538: https://www.ncbi.nlm.nih.gov/bioproject/PRJNA694538. All other data are available at https://doi.org/10.17605/osf.io/hafg6.

## APPENDIX A

(See Figures A1, A2, A3, A4, Table A1).

**Table A1:** Mean and standard error of larval and adult locomotion.

Treatment	Length (cm)	Speed (cm s^−1^)	Walk (times)	Jump (times)	Move (times)
Control	5.48 ± 0.18	0.032 ± 0.008	3.3 ± 0.4	0.5 ± 0.1	75.9 ± 6.4
−T1	5.63 ± 0.15	0.031 ± 0.001	4.0 ± 0.9	0.4 ± 0.1	59.0 ± 9.0
−T2	4.93 ± 0.27	0.026 ± 0.002	2.6 ± 0.6	0.7 ± 0.2	70.9 ± 9.8
−T3	5.86 ± 0.40	0.038 ± 0.005	3.5 ± 0.8	0.4 ± 0.1	97.8 ± 12.5
Antibiotic‐treated	5.76 ± 0.20	0.033 ± 0.005	3.6 ± 0.4	0.6 ± 0.2	69.7 ± 6.5
+T1	5.63 ± 0.35	0.030 ± 0.002	2.7 ± 0.6	0.6 ± 0.1	75.4 ± 11.6
+T2	5.83 ± 1.30	0.036 ± 0.008	4.5 ± 1.0	1.0 ± 0.2	69.3 ± 12.4
+T3	5.81 ± 1.30	0.032 ± 0.007	3.5 ± 0.8	0.3 ± 0.1	64.5 ± 10.7
Gut‐restoration	5.55 ± 0.22	0.031 ± 0.002	2.8 ± 0.3	0.5 ± 0.1	73.1 ± 6.2
+TR1	5.42 ± 1.21	0.030 ± 0.007	2.9 ± 0.7	0.8 ± 0.2	62.8 ± 9.3
+TR2	4.50 ± 1.01	0.024 ± 0.005	3.0 ± 0.7	0.3 ± 0.1	86.1 ± 13.2
+TR3	6.73 ± 1.50	0.038 ± 0.008	2.6 ± 0.6	0.6 ± 0.1	70.5 ± 9.3
